# Reasons for readmission after hospital discharge in patients with chronic diseases—Information from an international dataset

**DOI:** 10.1371/journal.pone.0233457

**Published:** 2020-06-30

**Authors:** Hans-Peter Brunner-La Rocca, Carol J. Peden, John Soong, Per Arne Holman, Maria Bogdanovskaya, Lorna Barclay

**Affiliations:** 1 Department of Cardiology, Maastricht University Medical Center, Maastricht, The Netherlands; 2 Center for Health System Innovation, Keck Medicine of USC, Los Angeles, California, United States of America; 3 NIHR CLAHRC for Northwest London Team, Imperial College London, Chelsea and Westminster Hospital NHS Foundation Trust, London, United Kingdom; 4 Department of Patients safety and Research, Lovisenberg Diaconal Hospital, Oslo, Norway; 5 Dr Foster Telstra Health, London, United Kingdom; Karolinska Institutet, SWEDEN

## Abstract

**Background:**

Chronic diseases are increasingly prevalent in Western countries. Once hospitalised, the chance for another hospitalisation increases sharply with large impact on well-being of patients and costs. The pattern of readmissions is very complex, but poorly understood for multiple chronic diseases.

**Methods:**

This cohort study of administrative discharge data between 2009–2014 from 21 tertiary hospitals (eight USA, five UK, four Australia, four continental Europe) investigated rates and reasons of readmissions to the same hospital within 30 days after unplanned admission with one of the following chronic conditions; heart failure; atrial fibrillation; myocardial infarction; hypertension; stroke; chronic obstructive pulmonary disease (COPD); bacterial pneumonia; diabetes mellitus; chronic renal disease; anaemia; arthritis and other cardiovascular disease. Proportions of readmissions with similar versus different diseases were analysed.

**Results:**

Of 4,901,584 admissions, 866,502 (17.7%) were due to the 12 chronic conditions. In-hospital, 43,573 (5.0%) patients died, leaving 822,929 for readmission analysis. Of those, 87,452 (10.6%) had an emergency 30-day readmission, rates ranged from 2.8% for arthritis to 18.4% for COPD. One third were readmitted with the same condition, ranging from 53% for anaemia to 11% for arthritis. Reasons for readmission were due to another chronic condition in 10% to 35% of the cases, leaving 30% to 70% due to reasons other than the original 12 conditions (most commonly, treatment related complications and infections). The chance of being readmitted with the same cause was lower in the USA, for female patients, with increasing age, more co-morbidities, during study period and with longer initial length of stay.

**Conclusion:**

Readmission in chronic conditions is very common and often caused by diseases other than the index hospitalisation. Interventions to reduce readmissions should therefore focus not only on the primary condition but on a holistic consideration of all the patient’s comorbidities.

## Introduction

Chronic diseases are increasingly prevalent in Western countries due to population aging and better treatment of underlying conditions. Once hospitalised, the chance for another hospitalisation increases sharply. The wide variation in readmission rates suggests that a significant proportion is avoidable [1]. The US Hospital Readmissions Reduction Program requires Centers for Medicare and Medicaid Services to reduce payments to hospitals with excess readmissions [2].

Readmissions pose a significant burden to patients and healthcare systems as they create significant mortality and morbidity [3]. The latter is not only important regarding well-being, but also has an important economic impact in form of costly hospitalisations [4]. Therefore, one important aim in treating chronic diseases is to prevent readmissions. The high risk of readmission has been shown for different chronic diseases [5–7] and after diverse interventions such as hip replacement, hip fracture, otolaryngology, bypass or general surgery [8–12]. The reasons for readmission after an intervention are often not primarily related to the intervention itself, but to the underlying comorbid conditions. Thus, chronic diseases may play an important role in readmission risk, independently of the reason for the initial hospitalisation [13].

Chronic diseases usually do not occur in isolation. Most patients with chronic disease have multiple diseases [14]. They may influence each other, and treatment for one disease may adversely impact the other. Hospital quality also impacts readmission rates [15]. Therefore, the pattern of readmissions may be very complex. Because of the interactions between disease and treatment, knowing patterns of readmissions related to different chronic diseases may improve the understanding of this important problem and reveal new treatment modalities for these patients. Unfortunately, most previous studies focused on single disease presentation at index hospitalisation and did not investigate the interplay between different diseases [16].

Therefore, we aimed to investigate reasons for readmission in patients with an index hospitalisation for multiple chronic conditions and three acute diseases usually related to an underlying chronic condition. We used data from an international dataset created from hospitals around the world through the Global Comparators project [17]. Our hypothesis was that reasons for readmission are often different from the index hospitalisation, irrespective of the underlying condition, and that this complexity increases with age and comorbidities. We also wanted to determine if the result changed over time, to what extent the results were influenced by in-hospital mortality, and if there were country specific differences.

## Methods

We collected electronic inpatient records from administrative discharge data provided by each of the 21 participating hospitals (eight from USA, five from UK, four from Australia, one each from Belgium, Denmark, Italy and the Netherlands; the latter four are combined as European centres; S1 Table), integrated into a uniform dataset as described elsewhere [17]. Institutional regulatory boards waived the requirement of Informed consent by patients and data were fully anonymised for the purpose of this analysis. For the present analysis, we used data from six complete calendar years, 2009–2014. All hospitals were major teaching hospitals. We excluded data before (i.e. 2007–2008) and after (i.e. 2015 and 2016) this period because many centres did not provide data for those periods to avoid selection bias. Between 2011–2013, complete data are available in all centres; in the two years before data from four hospitals (three in USA, one European) and in 2014 data from two UK hospitals were missing. Some hospitals reported in a previous publication [18] were excluded due to data quality issues, some missing codes and linkage issues from one year to the next.

We included records containing information on age, sex, country, principal diagnosis code (International Classification of Diseases, ICD-9 or ICD-10 [ICD-10_CM, ICD-10 AM]), Clinical Classification Software (CCS) group (Agency for Healthcare Quality and Research’s CCS groups (AHRQ CCS)) as described previously, [17] admission date, discharge date (date of death if admission ended in death) and in-hospital death. Each record was assigned a comorbidity score according to a modified version of the Elixhauser index [19], which is based on 32 conditions identified by secondary diagnoses codes [20]. Admissions were assigned to one of 259 diagnostic groups based on the primary diagnosis field (AHRQ CCS). Both bespoke chronic diseases (ICD-classification) and CCS codes were used to define the following chronic conditions as index hospitalisations: heart failure (HF); atrial fibrillation (AF); myocardial infarction (AMI); hypertension; stroke; chronic obstructive pulmonary disease (COPD); bacterial pneumonia; diabetes mellitus; chronic renal disease; anaemia; arthritis and other cardiovascular disease (see S2 Table). We included three acute conditions, i.e. stroke, AMI and bacterial pneumonia; AMI because it represents the most important event of an important underlying chronic condition, i.e. coronary artery disease, and pneumonia because it is often related to underlying chronic lung diseases, particularly COPD.

We excluded records corresponding to planned day-cases. Due to the difficulty in some countries in distinguishing patients admitted for observation only from those admitted as inpatients, we also excluded short-term emergency admissions with length of stay (LOS) less than two nights and no surgery.

Rates and reasons for readmissions within 30 days were analysed, focusing on the comparison between similar and different reasons for readmission compared with the index hospitalisation. Descriptive comparisons were made for each of the above-mentioned diseases. For a better illustration, Sankey diagrams are used to show the readmission patterns for each of these conditions [21]. Readmissions could only be identified when readmission was to the same hospital. For the analysis investigating readmission rates, in-hospital deaths were not considered. We allowed for a three-month window at the end of the time-period to ensure capturing the majority of readmissions for each hospital. For consistency across countries, readmissions with discharge after a three-month window were not counted, which accounted for <0.5% of the cases.

We further compared the readmission distributions for each of the 12 diseases, given by the proportion of readmissions to each of the 12 conditions or any CCS group. We calculated the Hellinger distance [22] between each pair, to identify clusters within the readmission patterns of the 12 diseases. By definition, the Hellinger distance is between zero and one, where zero means that patterns are identical, the larger the distance the more dissimilar the patterns.

To investigate the proportion of readmissions with similar versus different diseases we performed logistic regression with the outcome: readmission with the same versus any other condition. The model was adjusted for age, sex, year of discharge, comorbidity score, country, chronic condition and LOS. We applied a square root transformation to LOS as data are highly skewed to the right. Variables were considered significant when p<0.05. All data analyses were done using R, v. 3.4.2 [23].

## Results

**Fig 1:**
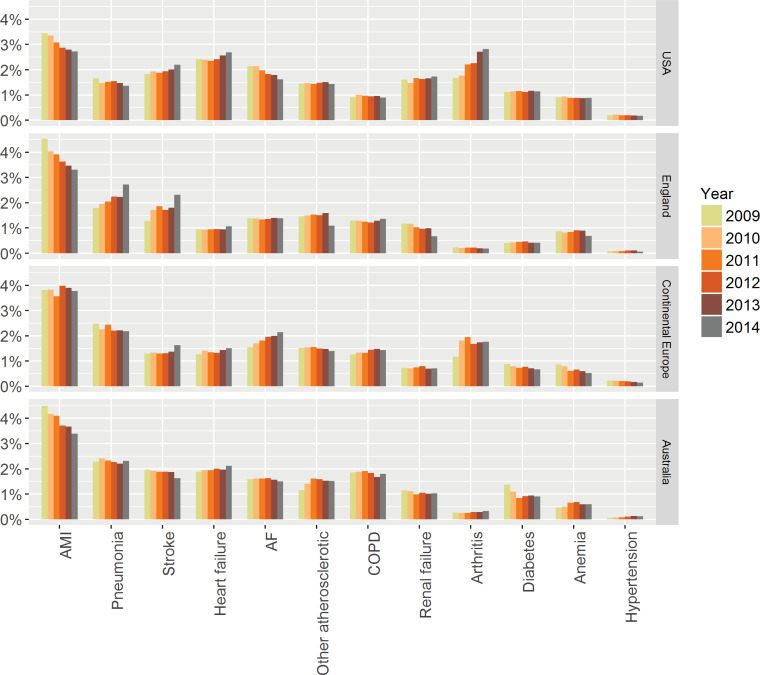
Proportion of admissions over time for the 12 chronic conditions in different countries. Abbreviations: AF atrial fibrillation; AMI acute myocardial infarction; COPD chronic obstructive pulmonary disease.

Out of 4,901,586 admissions, 866,502 (17.7%) were due to the 12 bespoke conditions as primary diagnosis (UK n = 207,748, 15.4%; continental Europe n = 187,182, 17.7%; Australia n = 161,171, 18.0%; USA n = 310,401, 19.4% USA). The patient characteristics are shown in Table 1. Patients in the USA were younger compared with the other countries, had a shorter LOS and more co-morbidities recorded. There were significant differences in baseline characteristics between the different conditions. Average LOS varied by a factor of almost three between the different conditions and differed between countries (Table 1). Table 2 shows the most common CCS groups / chronic conditions by number of admissions, indicating that the selected conditions are among the most important reasons for hospitalisation. Fig 1 provides an overview of the reasons of admission in the different countries over time.

**Table 1:** Patient characteristics.

	Average age	Average number of comorbidities	% male	Average length of stay (LOS)	Median LOS
**Overall**	65.4	2.29	56.6	7.5	4
**Country**					
Australia	68.4	1.81	58.1	8.5	4
Continental Europe	66.6	1.38	57.8	7.7	5
England	66.3	1.98	58.6	9.3	5
USA	62.5	3.30	53.6	5.8	4
**Chronic condition**					
AF	65.6	1.80	57.0	4.5	3
AMI	66.9	2.26	69.9	5.7	3
Anemia	52.4	1.68	45.3	6.2	4
Arthritis	63.9	1.46	42.1	4.3	3
COPD	68.6	1.77	51.0	7.6	5
Diabetes	52.4	2.20	56.5	7.8	4
Heart failure	70.6	3.57	55.5	8.9	6
Hypertension	62.4	1.49	43.3	4.9	3
Other cardiovascular	67.7	2.10	60.9	8.5	4
Pneumonia	64.2	2.31	53.3	8.9	6
Renal failure	63.1	3.06	56.7	8.5	5
Stroke	68.5	2.53	50.3	12.7	6

Abbreviations: AF atrial fibrillation; AMI acute myocardial infarction; COPD chronic obstructive pulmonary disease

**Table 2:** Most common CCS groups and chronic conditions as primary cause of initial hospitalization.

Condition group	Volume	Percentage
CCS group Liveborn	231377	4.72%
Chronic condition AMI	175437	3.58%
CCS group Complication of device, implant or graft	115533	2.36%
Chronic condition Pneumonia	97496	1.99%
CCS group Other complications of birth, puerperium affecting management of mother	90551	1.85%
Chronic condition Stroke	86189	1.76%
Chronic condition Heart failure	84963	1.73%
Chronic condition AF	82723	1.69%
CCS group Complications of surgical procedures or medical care	80353	1.64%
CCS group Spondylosis, intervertebral disc disorders, other back problems	75086	1.53%
Chronic condition Other cardiovascular disease	72414	1.48%
CCS group Other nervous system disorders	66199	1.35%
CCS group Biliary tract disease	65289	1.33%
Chronic condition COPD	63450	1.29%
CCS group Secondary malignancies	61933	1.26%
CCS group Urinary tract infections	61488	1.25%
CCS group Residual codes, unclassified	60878	1.24%
Chronic condition Arthritis	59969	1.22%
CCS group Septicemia (except in labour)	59506	1.21%
Chronic condition Chronic renal failure	57209	1.17%
CCS group Skin and subcutaneous tissue infections	56959	1.16%
CCS group Trauma to perineum and vulva	52616	1.07%
CCS group Normal pregnancy and/or delivery	51040	1.04%
CCS group Maintenance chemotherapy, radiotherapy	50700	1.03%
CCS group Fracture of upper limb	49437	1.01%
CCS group Foetal distress and abnormal forces of labour	48947	1.00%
CCS group Rehabilitation care, fitting of prostheses, and adjustment of devices	46808	.95%
CCS group Fracture of lower limb	46628	.95%
CCS group Other and unspecified benign neoplasm	45375	.93%
CCS group Epilepsy, convulsions	44791	.91%
CCS group Osteoarthritis	44689	.91%
CCS group Abdominal hernia	44214	.90%
CCS group Other connective tissue disease	44197	.90%
CCS group Other complications of pregnancy	44190	.90%
Chronic condition Diabetes	41136	.84%
Chronic condition Anemia	38163	.78%
CCS group Heart valve disorders	38089	.78%
CCS group Affective disorders	37311	.76%
CCS group Fracture of neck of femur (hip)	37177	.76%
CCS group Other fractures	36657	.75%
CCS group Appendicitis and other appendiceal conditions	36167	.74%
CCS group Other gastrointestinal disorders	34775	.71%
CCS group Other perinatal conditions	34214	.70%
CCS group Crushing injury or internal injury	33283	.68%
CCS group Other upper respiratory disease	32306	.66%
CCS group Cancer of bronchus, lung	31956	.65%
CCS group Gastrointestinal haemorrhage	31834	.65%
CCS group Abdominal pain	31453	.64%
CCS group Cancer of breast	31219	.64%
CCS group Intracranial injury	29403	.60%
CCS group Intestinal obstruction without hernia	29162	.59%
CCS group Fluid and electrolyte disorders	28882	.59%
CCS group Other nutritional, endocrine, and metabolic disorders	28152	.57%
CCS group Intestinal infection	27751	.57%
CCS group Pancreatic disorders (not diabetes)	27448	.56%
CCS group Calculus of urinary tract	27337	.56%
CCS group Acute bronchitis	27261	.56%
CCS group Nonspecific chest pain	26377	.54%
CCS group Other screening for suspected conditions	24198	.49%
CCS group Asthma	24019	.49%

Abbreviations: AF atrial fibrillation; AMI acute myocardial infarction; CCS Clinical Classification Software (definition see Methods); COPD chronic obstructive pulmonary disease

**Fig 2:**
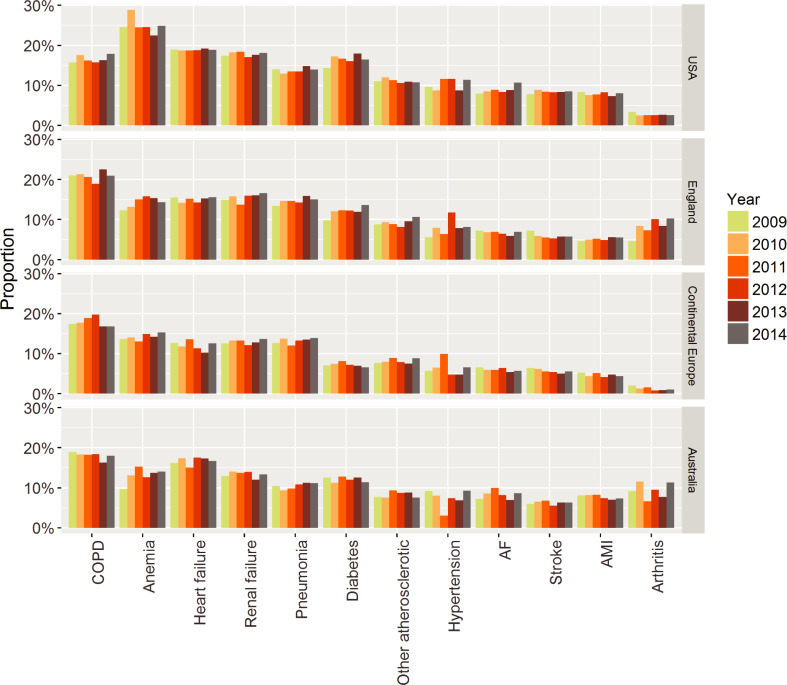
Proportion of readmissions over time in different countries for each of the 12 chronic conditions. Abbreviations: AF atrial fibrillation; AMI acute myocardial infarction; COPD chronic obstructive pulmonary disease.

**Fig 3:**
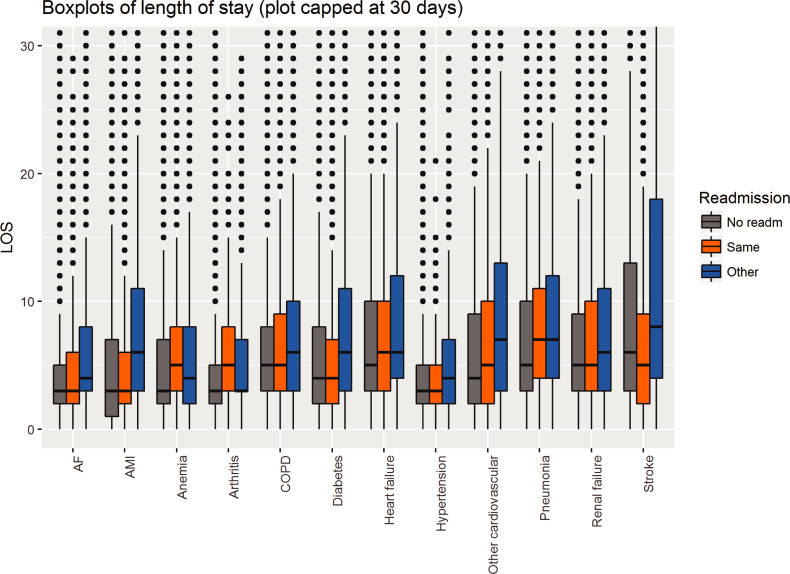
**Length of stay (LOS) for the index hospitalisation of the chronic conditions in those that were not readmitted (grey), readmitted with the same condition (orange) and with another condition (blue).** Abbreviations: AF atrial fibrillation; AMI acute myocardial infarction; COPD chronic obstructive pulmonary disease.

Overall, 43,573 (5.0%) patients died in-hospital with significant differences between the conditions (Table 3), leaving 822,929 admissions for readmission analysis. Of those, 87,452 (10.6%) had an emergency readmission within 30 days (Table 3). Readmission rate was highest in the USA (19.0%), followed by Australia (17.6%), England (16.5%) and continental Europe (14.6%). No major changes were seen over time, but there were some differences between the countries (Fig 2). Apart from anaemia and COPD, more than half of the readmissions were due to diseases other than the initial hospitalisation (Table 3). Interestingly, average LOS for the initial admission was comparable between patients that were not readmitted and were readmitted with the same condition in all 12 conditions but significantly longer in those readmitted due to another condition (Fig 3). Though the extent of this increase in LOS varied, it was seen in basically all conditions.

**Table 3:** Rate of in-hospital mortality, 30-day readmissions, percentage of conditions of readmissions and total events (combination of readmissions and deaths) in relation to the reason for index hospitalisation.

						% of all readmissions				
Condition	Total admissions	# of deaths	In-hospital mortality	Total discharges	30-day readmissions	Same condition	Other chronic condition	Other condition	Total events	Percentage events
Pneumonia	97,496	9,959	10.2%	87,537	11,507 (13.1%)	24.1%	20.5%	55.4%	21,466	22.0%
Heart failure	84,963	5,078	6.0%	79,885	13,333 (16.7%)	42.1%	22.7%	35.2%	18,411	21.7%
COPD	63,450	2,456	3.9%	60,994	11,231 (18.4%)	51.9%	17.9%	30.2%	13,687	21.8%
Renal failure	57,209	3,101	5.4%	54,108	8,579 (15.9%)	18.4%	18.8%	62.8%	11,680	20.4%
Anemia	38,163	568	1.5%	37,595	6,818 (18.1%)	52.9%	10.2%	36.9%	7,386	19.4%
Stroke	86,189	11,105	12.9%	75,084	5,134 (6.8%)	26.8%	14.8%	58.4%	16,239	18.8%
Diabetes	41,136	580	1.4%	40,556	5,310 (13.1%)	42.6%	13.8%	43.6%	5,890	14.3%
Other cardiovascular	72,414	3,894	5.4%	68,520	6,433 (9.4%)	21.2%	17.6%	61.2%	10,327	14.3%
Hypertension	7,353	63	0.9%	7,290	620 (8.5%)	22.9%	35.7%	41.4%	683	9.3%
AMI	175,437	5,374	3.1%	170,063	10,659 (6.3%)	27.4%	27.4%	45.2%	16,033	9.1%
AF	82,723	1,326	1.6%	81,397	6,169 (7.6%)	32.2%	26.3%	41.5%	7,495	9.1%
Arthritis	59,969	69	0.1%	59,900	1,659 (2.8%)	10.9%	17.4%	71.7%	1,728	2.9%
Total	866,502	43,573	5.0%	822,929	87,452 (10.6%)	33.9%	19.9%	46.2%	131,025	15.1%

Abbreviations: AF atrial fibrillation; AMI acute myocardial infarction; COPD chronic obstructive pulmonary disease

**Fig 4:**
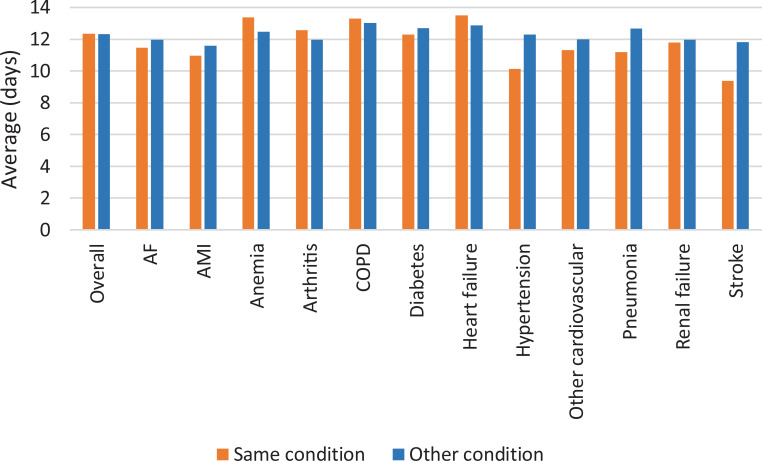
Average time to readmission overall and per original condition, depending on if readmission was due to the same or another condition. Abbreviations: AF atrial fibrillation; AMI acute myocardial infarction; COPD chronic obstructive pulmonary disease.

Overall, the average and median time to readmission did not differ between readmission due to the same or another condition (Fig 4). The median time until readmission was 11 days for both the same and another condition. Average time until readmission did not vary much between the 12 conditions with only hypertension and stroke having considerably shorter time to readmission for the same condition (Fig 4).

**Fig 5:**
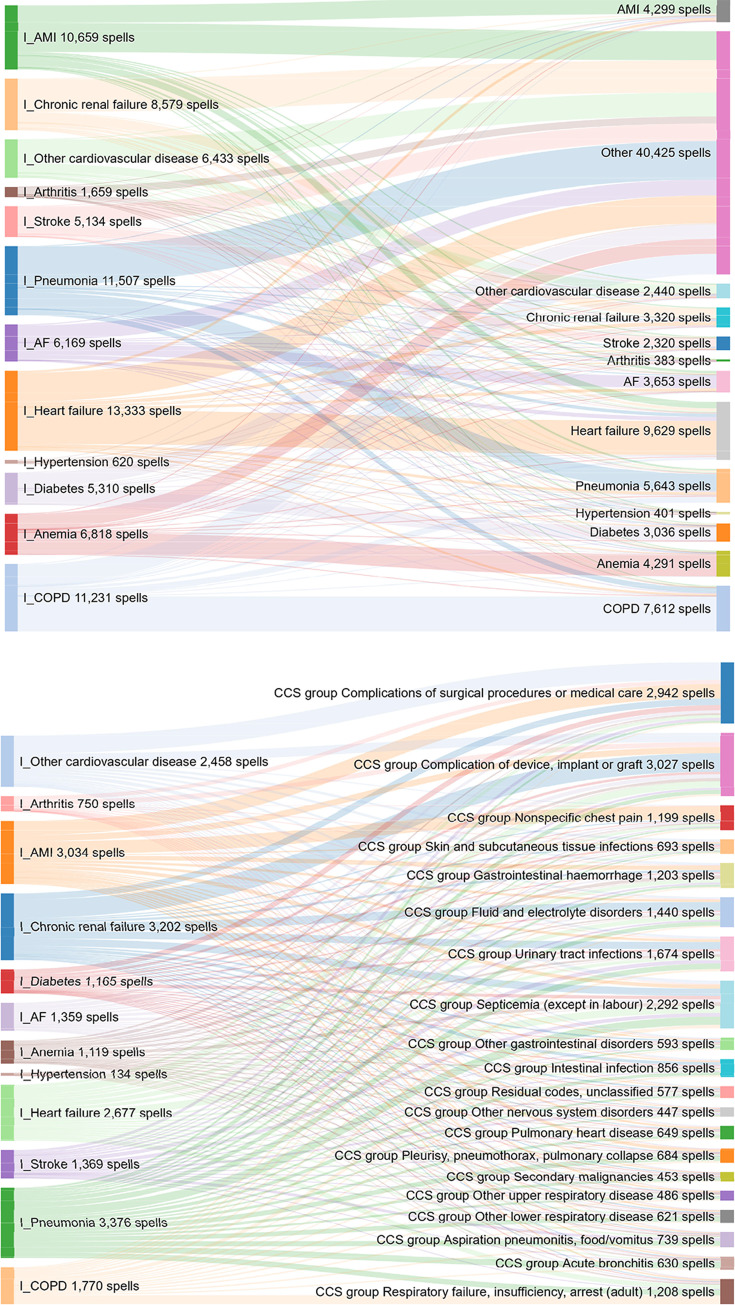
**Link between original condition of admission (left side) and cause of readmission (right side) for each chronic condition.** B provides information about all other causes in A. These figures (Supporting information: Interacting Fig 1A and 1B) can be found online by clicking on the figures to provide a dynamic depiction of the different causes of readmission for each chronic condition. Abbreviations: AF atrial fibrillation; AMI acute myocardial infarction; COPD chronic obstructive pulmonary disease.

The specific reasons for readmission of the 12 conditions are depicted in Fig 5A and 5B. These figures show that the causes of readmissions for a reason other than the same condition are diverse for all conditions. Regarding other causes than the 12 conditions, complications related to procedures / care during the initial hospitalisation or related to devices / implants are the most common ones, followed by infections. However, there was a large diversity of other causes (Fig 5B).

**Fig 6:**
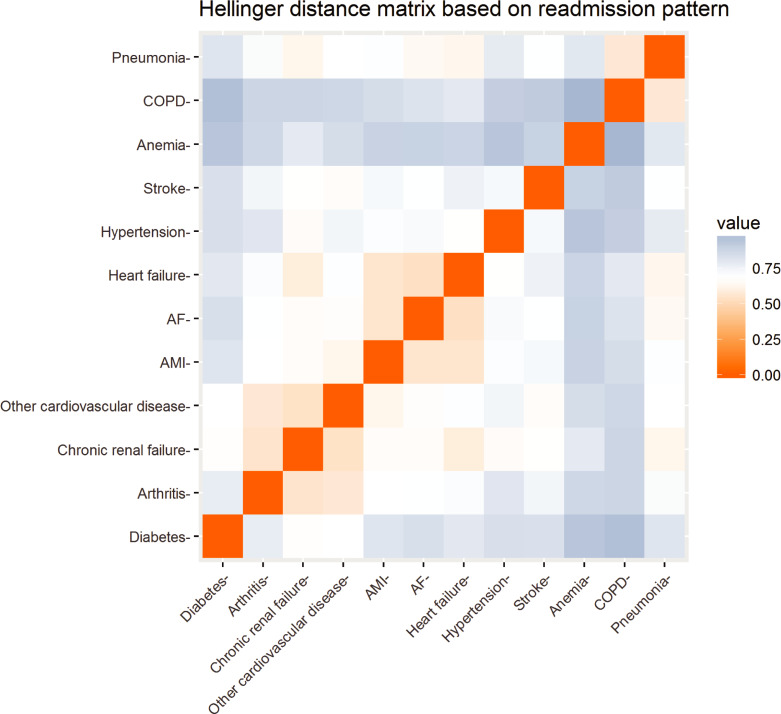
Readmission pattern for each of the chronic conditions (Hellinger distance matrix). Abbreviations: AF atrial fibrillation; AMI acute myocardial infarction; COPD chronic obstructive pulmonary disease.

In addition to readmission for the same reason (Hellinger distance = 0), several weak clusters (light orange) were identified, such as pneumonia and COPD; HF, MI and AF; other cardiovascular, chronic renal failure and arthritis (Fig 6). COPD and anaemia had the greatest distance from each other, and anaemia was far away from all other conditions. Anaemia was also the condition with the highest proportion of readmissions with the same condition (Table 3).

**Fig 7:**
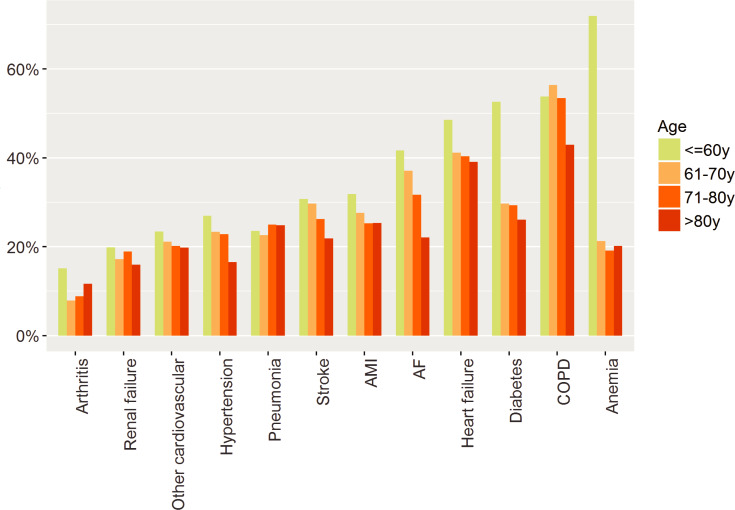
Readmission rates with the same condition by age for each of the chronic conditions. Abbreviations: AF atrial fibrillation; AMI acute myocardial infarction; COPD chronic obstructive pulmonary disease.

Various factors made readmission with the same condition more or less likely (Table 4). Patients with the initial hospitalisation due to COPD, HF, anaemia, and diabetes were most likely admitted with the same condition. In the USA, readmission due to the same cause was less likely. The chance of being readmitted due to the same cause was increased in male patients, with lower age, with less co-morbidities, at the beginning of the study period and with shorter LOS during initial admission. The effect of age was particularly evident in patients aged <60 years, and less evident at older ages, but the effect of age was not the same for all chronic conditions (Fig 7).

**Table 4:** Odds ratios (OR) of multivariable regression for probability of readmission with the same condition as initial hospitalisation.

	OR (95%-confidence interval)
Chronic Condition: Arthritis	1.000 (Reference)
Chronic Condition: AF	4.257*** (3.610, 5.021)
Chronic Condition: AMI	3.366*** (2.863, 3.958)
Chronic Condition: Anaemia	7.328*** (6.219, 8.634)
Chronic Condition: Renal failure	2.021*** (1.713, 2.386)
Chronic Condition: COPD	9.460*** (8.057, 11.109)
Chronic Condition: Diabetes	5.468*** (4.632, 6.454)
Chronic Condition: Heart failure	7.774*** (6.624, 9.122)
Chronic Condition: Hypertension	2.485*** (1.943, 3.179)
Chronic Condition: Other cardiovascular	2.397*** (2.027, 2.834)
Chronic Condition: Pneumonia	2.934*** (2.494, 3.451)
Chronic Condition: Stroke	3.852*** (3.255, 4.559)
Country: Australia	1.000 (Reference)
Country: England	.968 (.922, 1.017)
Country: Continental Europe	.979 (.935, 1.026)
Country: USA	.728*** (.698, .760)
Female	.954*** (.926, .983)
Age	.984*** (.983, .984)
Comorbidity score	.986*** (.985, .987)
Year	.987*** (.978, .996)
LOS (sqrt days)	.870*** (.859, .881)
C-statistic	.695
Observations	87,452

*p < .1

**p < .05

***p < .01

Abbreviations: AF atrial fibrillation; AMI acute myocardial infarction; COPD chronic obstructive pulmonary disease

## Discussion

In this large multinational cohort of hospital admissions with various chronic conditions, readmissions within 30 days were common and the reason for readmission differed from the index admission in more than 50% of the cases. This is true among all participating countries and all conditions investigated, despite significant differences between countries and conditions. This is the most comprehensive study reporting readmission rates for a broad spectrum of chronic conditions in different countries. It highlights the extent and the complexity of the problem as well as the need for focus not only on the disease that caused the initial hospitalisation, but also on other frequently related conditions. This stresses the need to approach readmission prevention from a holistic consideration of all the patient’s comorbidities.

When a person-centred approach is to the whole patient, readmission rates may be reduced [24]. It is surprising that there is not more focus on this, given that the number of comorbidities is associated with increased mortality and an increased readmission rate, both early and later after discharge [25, 26]. If comorbidities primarily increase the susceptibility of deteriorating with the same condition or simply increase the risk of manifestation of other diseases has not yet been properly investigated, but comorbidities clearly increase the complexity of the condition [27]. In patients with an index hospitalisation of COPD, readmission due to other causes was accompanied with significantly higher mortality than readmission due to COPD [28]. Therefore, the patterns accompanied with the highest risks of readmission should be more adequately investigated and addressed to prevent early events. Readmission is a multifactorial phenomenon constructed as a quality measure in many countries, with contributions from underlying disease and comorbidities (as found in our data), patient factors (e.g. psychosocial resilience), healthcare worker effects, environmental and social determinants of health, organisational and healthcare system factors [15, 16, 29, 30]. In addition, the organisation of care post-discharge, such as timeliness of follow-up, coordination with primary care, and quality of medication management may significantly influence readmission rate [31, 32]. Preventable readmission is what we should be focusing on, but consensus on how to achieve this is poor [33]. Our data suggest that the focus on and treatment of the index condition only, as currently done in many institutions is not sufficient. Thus, our results explain, at least in part, why measures to prevent rehospitalisation have had only a limited impact on the readmission rate, and that successful programs must address multiple aspects of patient care [26, 30].

Disease management programs are advocated in chronic conditions such as HF or COPD after discharge to reduce the readmission rate [34–36]. Although there is no uniform way to provide managed care, the majority of such programs improve outcome as shown in several meta-analyses [37–39]. The rather broad approach to care of these programs might result in reduction of readmission not only due to the index disease. The clustering of diseases with some clinical validity found in our analysis might allow such assumption. This notion remains speculative, however, until properly tested.

Although the spectrum of causes for readmissions was broad, independently of the cause of the initial admission, there are some conditions that were more common. Thus, myocardial infarction or atrial fibrillation may lead to HF, (treatment of) HF may cause renal failure, and patients with COPD are more susceptible to acquire pneumonia. There were, however, causes that were seen in basically all conditions. Many patients had infections as cause of readmission, in line with previous reports [28]. It suggests that such patients are vulnerable early after discharge to acquire infectious diseases or infections may be acquired during hospitalisation (e.g. urinary tract infections due to urine catheters; hospital acquired pneumonia). Additionally, the susceptibility of these patients may be higher than during more stable conditions. Using sufficient hygiene measures such infections might in part be preventable if the general awareness is improved. Education of patients and their families about the increased risk of infection may also be important to aid rapid diagnosis and treatment.

Another important readmission group is related to complications of medical procedures or devices. Although it is not well investigated to what extent such complications are preventable in the setting of chronic conditions, it may be worthwhile to specifically investigate such complications and to test preventive measures to reduce the number. Often, such measures are only taken in the context of surgery and/or invasive procedures, but not as a general routine in patients with chronic conditions.

There was a small, but significant trend for less readmissions with the same condition as the index hospitalisation over time. The aging population and the fact that comorbidities are increasingly frequent at higher age may be accountable for this finding. Age and comorbidities are included in the regression equation as well, which may highlight this, but may have diminished the overtime effect. Therefore, we may expect that the complexity of patients admitted with chronic conditions is increasing in the future as the population ages further. It may also be explained as less complex procedures, and less severe conditions, are treated more in the outpatient setting. Therefore, the increasing complexity of hospitalised patients needs to be considered for future planning of hospital care of chronic diseases.

### Limitations

There are several limitations to our study. Importantly, we used administrative data, capturing only what was coded. Coding is not uniform and may vary depending on diagnosis related group, degree of in-hospital testing, national reimbursement incentives, physician and coder training and institutional variation [40, 41]. Some of these factors may explain why patients in the USA had a higher comorbidity score although they were younger and had a shorter LOS. The higher comorbidities in US patients could also reflect issues of access and late presentation [42]. Direct comparison between countries, and also between institutions, regarding the absolute burden of disease is not possible. Also, risk adjustment considering all factors influencing readmissions is limited. However, the purpose of this study was to investigate the associations between index hospitalisation and readmission. As this only relates to the primary diagnosis, coding has a much lower impact. Moreover, the aim was to investigate the global picture of the problem, which is not significantly influenced by the limitation of coding. We did not compare single institutions with each other due to reasons of confidentiality given the limited number of participating hospitals, particularly in continental Europe. Administrative models may have limited discriminatory abilities, which raises concerns about the ability to standardise risk across hospitals to fairly compare hospital performance [16]. Our data suggest that previously insufficiently recognised factors related to the complexity of multimorbid patients need to be included to improve risk prediction and adjustment.

We included conditions that present with acute events (i.e. AMI, pneumonia, stroke) but are usually caused by a chronic underlying disease. Including these conditions might have influenced our results. However, readmission pattern of them were in line with the other more chronic conditions, supporting the assumption that the readmissions were mainly influenced by the underlying chronic condition rather than the acute event.

In addition, participating hospitals are academic centres and focus may differ between centres. Therefore, there may be an imbalance in the patient population and differences in the spectrum of patients in centres that did not participate. Also, readmission to a different hospital could not be recorded. Therefore, the true readmission rate is likely even higher than reported here although the disease specific readmission rates may be similar to those reported in the past (for example 7 vs 5% for heart failure) [43]. It might be speculated that readmission due to other causes than the initial admission is more likely to be treated in another hospital than the index admission and the absolute values must be interpreted with caution. Nevertheless, the consequence of these two factors would be an even higher readmission rate and a higher proportion of causes different than the initial admission, even more strongly supporting the conclusion of our study.

## Conclusions

Readmissions within 30 days after discharge are very common in patients with chronic conditions. These readmissions are, in more than 50% of patients, related to a different cause than the initial hospitalisation. Therefore, this study has significant implications, for clinicians, the hospital administrators and for policy makers. It highlights the importance of a global approach to the treatment and management of patients with chronic conditions. Focus on the chronic condition and the circumstances that led to the hospitalisation is not sufficient, and additional measures based on a more holistic approach to the individual patient should be taken to significantly reduce readmissions.

## Supporting information

S1 Table List of participating centres. (DOCX)

S2 Table ICD 9 and ICD 10 codes for chronic conditions. (DOCX)

S1 Fig A and B: Link between original condition of admission (left side) and cause of readmission (right side) for each chronic condition. B provides information about all other causes in A. Interactive online figures to provide a dynamic depiction of the different causes of readmission for each chronic condition.
(HTML)
