# Supplementary material for: Reasons for readmission after hospital discharge in patients with chronic diseases—Information from an international dataset
Source: PLoS One. 2020 Jun 30;15(6):e0233457. doi: 10.1371/journal.pone.0233457 (PMC7326238; doi:10.1371/journal.pone.0233457)
Supplement: S1 Table — (DOCX) [file pone.0233457.s001.docx]

Supplementary table 1 List of participating centres

| **Hospital name** | **City** | **Country** | **Contact** | **Website** |
| --- | --- | --- | --- | --- |
| Alfred Health | Melbourne | Australia | +61 3 90762000 | www.alfredhealth.org.au |
| Austin Health | Melbourne | Australia | +61 3 94965000 | www.austin.org.au |
| Melbourne Health | Melbourne | Australia | +61 3 93427000 | www.thermh.org.au |
| Monash Health | Melbourne | Australia | +61 3 95946666 | monashhealth.org |
| Universitair ziekenhuis Leuven | Leuven | Belgium | +32 16 332211 | www.uzleuven.be/en |
| Aalborg UH | Aalborg | Denmark | +45 97 666000 | aalborguh.rn.dk/service/english |
| Guy's and St Thomas' | London | England | +44 20 75895111 | www.imperial.ac.uk |
| Imperial College Healthcare | London | England | +44 20 33113311 | www.imperial.nhs.uk |
| Royal United Hospitals Bath | Bath | England | +44 1225 428331 | www.ruh.nhs.uk |
| University College London Hospitals | London | England | +44 20 34567890 | www.uclh.nhs.uk |
| University Hospitals Coventry & Warwickshire | Coventry | England | +44 24 76964000 | www.uhcw.nhs.uk |
| Academisch Ziekenhuis Maastricht | Maastricht | The Netherlands | +31 43 3876543 | www.mumc.nl |
| Humanitas Research Hospital | Milan | Italy | +39 02 82246250 | www.humanitas.it |
| Barnes-Jewish Hospital | St. Louis | USA | +1 314 7473000 | www.barnesjewish.org |
| Hackensack University Medical Center | Hackensack | USA | +1 844 4649355 | www.hackensackumc.org |
| Hospital of the University of Pennsylvania | Philadelphia | USA | +1 215 6624000 | www.pennmedicine.org |
| Huntsville Hospital | Huntsville | USA | +1 256 2651000 | www.huntsvillehospital.org |
| Keck Hospital of USC | Los Angeles | USA | +1 800 8722273 | www.keckmedicine.org |
| UC San Diego Medical Center | San Diego | USA | +1 858 6577000 | health.ucsd.edu |
| University of Texas Southwestern Medical Center | Dallas | USA | +1 214 6483111 | www.utsouthwestern.edu |
| Yale–New Haven Hospital | New Haven | USA | +1 203 6884242 | www.ynhh.org |
