# Supplementary material for: Reasons for readmission after hospital discharge in patients with chronic diseases—Information from an international dataset
Source: PLoS One. 2020 Jun 30;15(6):e0233457. doi: 10.1371/journal.pone.0233457 (PMC7326238; doi:10.1371/journal.pone.0233457)
Supplement: S2 Table — (DOCX) [file pone.0233457.s002.docx]

Supplementary table 2 ICD 9 and ICD 10 codes for chronic conditions

| **Chronic Condition** | **ICD 10** |  | **ICD 9** |  |
| --- | --- | --- | --- | --- |
|  |  |  |  |  |
| AMI | I20 | Angina pectoris | 410 | Acute myocardial infarction |
|  | I21 | Acute myocardial infarction | 411 | Other acute & subacute forms of ischemic heart disease |
|  | I22 | Subsequent myocardial infarction | 412 | Old myocardial infarction |
|  | I24 | Other acute ischaemic heart diseases | 413 | Angina pectoris |
|  | I251 | Atherosclerotic heart disease | 414 | Other forms of chronic ischemic heart disease |
|  | I252 | Old myocardial infarction | 41400 | Coronary atherosclerosis of unspecified type of vessel, native or graft |
|  | I253 | Aneurysm of heart | 41401 | Coronary atherosclerosis of native coronary artery |
|  | I255 | Ischaemic cardiomyopathy | 41403 | Coronary atherosclerosis of nonautologous biological bypass graft |
|  | I256 | Silent myocardial ischaemia | 41406 | Coronary atherosclerosis of native coronary artery of transplanted heart |
|  | I258 | Other forms of chronic ischaemic heart disease | 4141 | Aneurysm of heart |
|  | I259 | Chronic ischaemic heart disease, unspecified | 4142 | Chronic total occlusion of coronary artery |
|  |  |  | 4143 | Coronary atherosclerosis due to lipid rich plaque |
|  |  |  | 4144 | Coronary atherosclerosis due to calcified coronary lesion |
|  |  |  | 4148 | Other specified forms of chronic ischemic heart disease |
|  |  |  | 4149 | Chronic ischemic heart disease, unspecified |
|  |  |  | V4581 | Aortocoronary bypass status |
|  |  |  | V4582 | Percutaneous transluminal coronary angioplasty status |
|  |  |  |  |  |
| Anemia | D50 | Iron deficiency anaemia | 280 | Iron deficiency anemias |
|  | D51 | Vitamin Bâ‚â‚‚ deficiency anaemia | 281 | Other deficiency anemias |
|  | D52 | Folate deficiency anaemia | 282 | Hereditary hemolytic anemias |
|  | D53 | Other nutritional anaemias | 283 | Acquired hemolytic anemias |
|  | D55 | Anaemia due to enzyme disorders | 284 | Aplastic anemia |
|  | D56 | Thalassaemia | 285 | Other & unspecified anemias |
|  | D57 | Sickle-cell disorders |  |  |
|  | D58 | Other hereditary haemolytic anaemias |  |  |
|  | D59 | Acquired haemolytic anaemia |  |  |
|  | D60 | Acquired pure red cell aplasia [erythroblastopenia] |  |  |
|  | D61 | Other aplastic anaemias |  |  |
|  | D62 | Acute posthaemorrhagic anaemia |  |  |
|  | D63 | Anaemia in chronic diseases classified elsewhere |  |  |
|  | D64 | Other anaemias |  |  |
|  |  |  |  |  |
| Arthritis | M05 | Seropositive rheumatoid arthritis | 274 | Gout |
|  | M06 | Other rheumatoid arthritis | 7120 | Crystal arthropathies |
|  | M07 | Psoriatic and enteropathic arthropathies | 7121 | Chondrocalcinosis due to dicalcium phosphate crystals |
|  | M10 | Gout | 7122 | Chondrocalcinosis due to pyrophosphate crystals |
|  | M11 | Other crystal arthropathies | 7123 | Chondrocalcinosis, cause unspecified |
|  | M13 | Other arthritis | 7128 | Other specified crystal arthropathies |
|  |  |  | 7129 | Unspecified crystal arthropathy |
|  |  |  | 713 | Arthropathy associated with other disorders classified elsewhere |
|  |  |  | 714 | Rheumatoid arthritis & other inflammatory polyarthropathies |
|  |  |  | 715 | Osteoarthrosis & allied disorders |
|  |  |  | 7160 | Kaschin-Beck disease |
|  |  |  | 7161 | Traumatic arthropathy |
|  |  |  | 7162 | Allergic arthritis |
|  |  |  | 7163 | Climacteric arthritis |
|  |  |  | 7164 | Transient arthropathy |
|  |  |  | 7165 | Unspecified polyarthropathy or polyarthritis |
|  |  |  | 7166 | Unspecified monoarthritis |
|  |  |  | 7168 | Other specified arthropathy |
|  |  |  | 7169 | Unspecified arthropathy |
|  |  |  |  |  |
| Atrial fibrillation | I47 | Paroxysmal tachycardia | 427 | Cardiac dysrhythmias |
|  | I48 | Atrial fibrillation and flutter | 4270 | Paroxysmal supraventricular tachycardia |
|  | I49 | Other cardiac arrhythmias | 4271 | Paroxysmal ventricular tachycardia |
|  | R00 | Abnormalities of heart beat | 4272 | Paroxysmal tachycardia, unspecified |
|  |  |  | 4273 | Atrial fibrillation & flutter |
|  |  |  | 4276 | Premature beats |
|  |  |  | 4278 | Other specified cardiac dysrhythmias |
|  |  |  | 4279 | Cardiac dysrhythmia, unspecified |
|  |  |  | 7850 | Tachycardia, unspecified |
|  |  |  | 7851 | Palpitations |
|  |  |  |  |  |
| Chronic renal failure | E102 | Type 1 diabetes mellitus - With renal complications | 2494 | Secondary diabetes mellitus with renal manifestations |
|  | E112 | Type 2 diabetes mellitus - With renal complications | 2504 | Diabetes with renal manifestations |
|  | E122 | Malnutrition-related diabetes mellitus - With renal complications | 403 | Hypertensive renal disease |
|  | E132 | Other specified diabetes mellitus - With renal complications | 40413 | Hypertensive heart and chronic kidney disease, benign, with heart failure and chronic kidney disease stage V or end stage renal disease |
|  | E142 | Unspecified diabetes mellitus - With renal complications | 584 | Acute renal failure |
|  | I12 | Hypertensive renal disease | 585 | CHRONIC RENAL FAILURE (End 2005) |
|  | I131 | Hypertensive heart and renal disease with renal failure | 586 | Renal failure, unspecified |
|  | I132 | Hypertensive heart and renal disease with both (congestive) heart failure and renal failure | 7925 | Cloudy (hemodialysis) (peritoneal) dialysis effluent |
|  | I139 | Hypertensive heart and renal disease, unspecified | V420 | Kidney replaced by transplant |
|  | N17 | Acute renal failure | V451 | RENAL DIALYSIS STATUS |
|  | N18 | Chronic kidney disease | V56 | Aftercare involving intermittent dialysis |
|  | N19 | Unspecified kidney failure |  |  |
|  | Z49 | Care involving dialysis |  |  |
|  |  |  |  |  |
| COPD | J40 | Bronchitis, not specified as acute or chronic | 490 | Bronchitis, not specified as acute or chronic |
|  | J41 | Simple and mucopurulent chronic bronchitis | 491 | Chronic bronchitis |
|  | J42 | Unspecified chronic bronchitis | 492 | Emphysema |
|  | J43 | Emphysema | 494 | BRONCHIECTASIS (End 2000) |
|  | J44 | Other chronic obstructive pulmonary disease | 496 | Chronic airway obstruction, not elsewhere classified |
|  | J47 | Bronchiectasis |  |  |
|  |  |  |  |  |
| Diabetes | E09 (excluding E092) | Intermediate hyperglycaemia | 249 | Secondary diabetes mellitus |
|  | E10 (excluding E102) | Type 1 diabetes mellitus | 250 | Diabetes mellitus |
|  | E11 (excluding E112) | Type 2 diabetes mellitus | 7902 | ABN GLUCOSE TOLERAN TEST (End 2003) |
|  | E12 (excluding E122) | Malnutrition-related diabetes mellitus | 7915 | Glycosuria |
|  | E13 (excluding E132) | Other specified diabetes mellitus | 7916 | Acetonuria |
|  | E14 (excluding E142) | Unspecified diabetes mellitus | V4585 | Insulin pump status |
|  | R73 | Elevated blood glucose level | V5391 | Fitting and adjustment of insulin pump |
|  | R81 | Glycosuria | V6546 | Encounter for insulin pump training |
|  | R824 | Acetonuria |  |  |
|  |  |  |  |  |
| Heart failure | I110 | Hypertensive heart disease with (congestive) heart failure | 39891 | Rheumatic heart failure (congestive) |
|  | I130 | Hypertensive heart and renal disease with (congestive) heart failure | 40201 | Malignant hypertensive heart disease with heart failure |
|  | I42 | Cardiomyopathy | 40211 | Benign hypertensive heart disease with heart failure |
|  | I50 | Heart failure | 40291 | Unspecified hypertensive heart disease with heart failure |
|  |  |  | 40401 | Hypertensive heart and chronic kidney disease, malignant, with heart failure and with chronic kidney disease stage I through stage IV, or unspecified |
|  |  |  | 40411 | Hypertensive heart and chronic kidney disease, benign, with heart failure and with chronic kidney disease stage I through stage IV, or unspecified |
|  |  |  | 40491 | Hypertensive heart and chronic kidney disease, unspecified, with heart failure and with chronic kidney disease stage I through stage IV, or unspecified |
|  |  |  | 425 | Cardiomyopathy |
|  |  |  | 428 | Heart failure |
|  |  |  |  |  |
| Hypertension | I10 | Essential (primary) hypertension | 401 | Essential hypertension |
|  | I119 | Hypertensive heart disease without (congestive) heart failure | 4020 | Malignant hypertensive heart disease |
|  |  |  | 40200 | Malignant hypertensive heart disease without heart failure |
|  |  |  | 4021 | Benign hypertensive heart disease |
|  |  |  | 40210 | Benign hypertensive heart disease without heart failure |
|  |  |  | 4029 | Unspecified hypertensive heart disease |
|  |  |  | 40290 | Unspecified hypertensive heart disease without heart failure |
|  |  |  | 40400 | Hypertensive heart and chronic kidney disease, malignant, without heart failure and with chronic kidney disease stage I through stage IV, or unspecified |
|  |  |  | 40402 | Hypertensive heart and chronic kidney disease, malignant, without heart failure and with chronic kidney disease stage V or end stage renal disease |
|  |  |  | 40403 | Hypertensive heart and chronic kidney disease, malignant, with heart failure and with chronic kidney disease stage V or end stage renal disease |
|  |  |  | 4041 | BEN HYPERT HRT/RENAL DIS (Begin 1980 |
|  |  |  | 40410 | Hypertensive heart and chronic kidney disease, benign, without heart failure and with chronic kidney disease stage I through stage IV, or unspecified |
|  |  |  | 40412 | Hypertensive heart and chronic kidney disease, benign, without heart failure and with chronic kidney disease stage V or end stage renal disease |
|  |  |  | 40490 | Hypertensive heart and chronic kidney disease, unspecified, without heart failure and with chronic kidney disease stage I through stage IV, or unspecified |
|  |  |  | 40492 | Hypertensive heart and chronic kidney disease, unspecified, without heart failure and with chronic kidney disease stage V or end stage renal disease |
|  |  |  | 40493 | Hypertensive heart and chronic kidney disease, unspecified, with heart failure and chronic kidney disease stage V or end stage renal disease |
|  |  |  |  |  |
| Pneumonia | A202 | Pneumonic plague | 00322 | Salmonella pneumonia |
|  | A212 | Pulmonary tularaemia | 0203 | Primary pneumonic plague |
|  | A221 | Pulmonary anthrax | 0204 | Secondary pneumonic plague |
|  | A310 | Pulmonary mycobacterial infection | 0205 | Pneumonic plague, unspecified |
|  | A420 | Pulmonary actinomycosis | 0212 | Pulmonary tularemia |
|  | A430 | Pulmonary nocardiosis | 0221 | Pulmonary anthrax |
|  | A481 | Legionnaires disease | 0310 | Pulmonary diseases due to other mycobacteria |
|  | A78 | Q fever | 0391 | Pulmonary actinomycotic infection |
|  | B012 | Varicella pneumonia | 0521 | Varicella (hemorrhagic) pneumonitis |
|  | B052 | Measles complicated by pneumonia | 0551 | Postmeasles pneumonia |
|  | B250 | Cytomegaloviral pneumonitis | 0730 | Ornithosis with pneumonia |
|  | B583 | Pulmonary toxoplasmosis | 0830 | Q fever |
|  | B59 | Pneumocystosis | 1124 | Candidiasis of lung |
|  | B671 | Echinococcus granulosus infection of lung | 1140 | Primary coccidioidomycosis (pulmonary) |
|  | J12 | Viral pneumonia, not elsewhere classified | 1144 | Chronic pulmonary coccidioidomycosis |
|  | J13 | Pneumonia due to Streptococcus pneumoniae | 1145 | Pulmonary coccidioidomycosis, unspecified |
|  | J14 | Pneumonia due to Haemophilus influenzae | 11505 | Infection by Histoplasma capsulatum, pneumonia |
|  | J15 | Bacterial pneumonia, not elsewhere classified | 11515 | Infection by Histoplasma duboisii, pneumonia |
|  | J16 | Pneumonia due to other infectious organisms, not elsewhere classified | 11595 | Histoplasmosis, unspecified, pneumonia |
|  | J17 | Pneumonia in diseases classified elsewhere | 1304 | Pneumonitis due to toxoplasmosis |
|  | J18 | Pneumonia, organism unspecified | 1363 | Pneumocystosis |
|  | J850 | Gangrene and necrosis of lung | 480 | Viral pneumonia |
|  | J851 | Abscess of lung with pneumonia | 481 | Pneumococcal pneumonia [Streptococcus pneumoniae pneumonia] |
|  |  |  | 482 | Other bacterial pneumonia |
|  |  |  | 483 | PNEUMONIA: ORGANISM NEC (Begin 1980 |
|  |  |  | 485 | Bronchopneumonia, organism unspecified |
|  |  |  | 486 | Pneumonia, organism unspecified |
|  |  |  | 5130 | Abscess of lung |
|  |  |  | 5171 | Rheumatic pneumonia |
|  |  |  |  |  |
| Stroke | G46 | Vascular syndromes of brain in cerebrovascular diseases | 430 | Subarachnoid hemorrhage |
|  | I60 | Subarachnoid haemorrhage | 431 | Intracerebral hemorrhage |
|  | I61 | Intracerebral haemorrhage | 432 | Other & unspecified intracranial hemorrhage |
|  | I62 | Other nontraumatic intracranial haemorrhage | 4330 | BASILAR ARTERY OCCLUSION (End 1993) |
|  | I63 | Cerebral infarction | 43301 | Occlusion and stenosis of basilar artery with cerebral infarction |
|  | I64 | Stroke, not specified as haemorrhage or infarction | 4331 | CAROTID ARTERY OCCLUSION (End 1993) |
|  | I66 | Occlusion and stenosis of cerebral arteries, not resulting in cerebral infarction | 43311 | Occlusion and stenosis of carotid artery with cerebral infarction |
|  | I693 | Sequelae of cerebral infarction | 4332 | VERTEBRAL ART OCCLUSION (End 1993) |
|  | I694 | Sequelae of stroke, not specified as haemorrhage or infarction | 43321 | Occlusion and stenosis of vertebral artery with cerebral infarction |
|  |  |  | 4333 | MULT PRECEREB OCCLUSION (End 1993) |
|  |  |  | 43331 | Occlusion and stenosis of multiple and bilateral precerebral arteries with cerebral infarction |
|  |  |  | 4338 | PRECEREB OCCLUSION NEC (End 1993) |
|  |  |  | 43381 | Occlusion and stenosis of other specified precerebral artery with cerebral infarction |
|  |  |  | 4339 | PRECEREB OCCLUSION NOS (End 1993) |
|  |  |  | 43391 | Occlusion and stenosis of unspecified precerebral artery with cerebral infarction |
|  |  |  | 434 | Occlusion of cerebral arteries |
|  |  |  | 436 | Acute, but ill-defined, cerebrovascular disease |
|  |  |  | 437 | Other & ill-defined cerebrovascular disease |
|  |  |  |  |  |
| Other cardiovascular disease | I95 | Hypotension | 42682 | Long QT syndrome |
|  | I988 | Other specified disorders of circulatory system in diseases classified elsewhere | 440 | Atherosclerosis |
|  | I99 | Other and unspecified disorders of circulatory system | 4400 | Atherosclerosis of aorta |
|  | K55 | Vascular disorders of intestine | 4401 | Atherosclerosis of renal artery |
|  | M30 | Polyarteritis nodosa and related conditions | 4402 | ATHEROSCLEROS-EXTREMITY (Begin 1980 |
|  | M31 | Other necrotizing vasculopathies | 44020 | Atherosclerosis of native arteries of the extremities, unspecified |
|  | R030 | Elevated blood-pressure reading, without diagnosis of hypertension | 44021 | Atherosclerosis of native arteries of the extremities with intermittent claudication |
|  | R031 | Nonspecific low blood-pressure reading | 44022 | Atherosclerosis of native arteries of the extremities with rest pain |
|  | R58 | Haemorrhage, not elsewhere classified | 44023 | Atherosclerosis of native arteries of the extremities with ulceration |
|  | R943 | Abnormal results of cardiovascular function studies | 44029 | Other atherosclerosis of native arteries of the extremities |
|  |  |  | 4404 | Chronic total occlusion of artery of the extremities |
|  |  |  | 4408 | Atherosclerosis of other specified arteries |
|  |  |  | 4409 | Generalized and unspecified atherosclerosis |
|  |  |  | 441 | Aortic aneurysm |
|  |  |  | 442 | Other aneurysm |
|  |  |  | 443 | Other peripheral vascular disease |
|  |  |  | 444 | Arterial embolism & thrombosis |
|  |  |  | 446 | Polyarteritis nodosa & allied conditions |
|  |  |  | 447 | Other disorders of arteries & arterioles |
|  |  |  | 448 | Disease of capillaries |
|  |  |  | 458 | Hypotension |
|  |  |  | 4580 | Orthostatic hypotension |
|  |  |  | 4581 | Chronic hypotension |
|  |  |  | 4588 | Other specified hypotension |
|  |  |  | 4589 | Hypotension, unspecified |
|  |  |  | 459 | Other disorders of circulatory system |
|  |  |  | 4590 | Hemorrhage, unspecified |
|  |  |  | 45989 | Other specified disorders of circulatory system |
|  | V125 | HX-CIRCULATORY SYS DIS (End 1995) | 4599 | Unspecified circulatory system disorder |
|  | V151 | Personal history of surgery to heart and great vessels, presenting hazards to health | 557 | Vascular insufficiency of intestine |
|  | V421 | Heart replaced by transplant | 785 | Symptoms involving cardiovascular system |
|  | V432 | HEART REPLACEMENT NEC (End 2003) | 7859 | Other symptoms involving cardiovascular system |
|  | V434 | Blood vessel replaced by other means | 7943 | Nonspecific abnormal results of function study of cardiovascular system |
|  | V717 | Observation for suspected cardiovascular disease | 7962 | Elevated blood pressure reading without diagnosis of hypertension |
