# Supplementary material for: Reasons for readmission after hospital discharge in patients with chronic diseases—Information from an international dataset
Source: PLoS One. 2020 Jun 30;15(6):e0233457. doi: 10.1371/journal.pone.0233457 (PMC7326238; doi:10.1371/journal.pone.0233457)
Supplement: Attachment — Submitted filename: Rebuttal PLOS ONE.docx [file pone.0233457.s004.docx]

**Comments to by the reviewers**

Reviewer #1:

The study is relevant, interesting and well written. I think this paper has the ability for a better understanding of the treatment and management of patients with chronic conditions and the described high rate of about 17% of readmissions. The conclusions could be strengthened. However, the present study is significantly limited by the administrative nature of the data, the lack of robust risk adjustment and the imbalances in the patient population.

*We would like to thank the reviewer for carefully reading our manuscript and to provide positive feedback on it.*

*We agree with the reviewer that the study has important limitations that we also addressed in part in the limitation section of the original submission. We now added the fact that risk adjustment was limited and that there may be an imbalance in the patient population. This is inevitable to administrative data. The advantage of administrative data is that there is no preselection of patients, which is inevitable to most registries. For the purpose of the study, we are convinced that administrative data are useful.*

There are some major concerns and limitations which have to be to addressed:
- The authors should clarify whether the identified ICD codes were main or secondary diagnoses for readmission of the patients. This fact could potentially bias the observed findings.

*We agree with the reviewer. This is why we only used main diagnosis for readmission of patients to keep the potential bias as small as possible. We are aware that the coding may be subjective and not homogenous across all centres. This is addressed in the limitation section of the manuscript.*

- Why did the authors choose the 30 days as time point of emergency readmission?

*To some extent, this is obviously arbitrary. However, the reason for this is mainly twofold: a) reimbursement policy in the USA is related to the 30-day readmission rate. The result of this fact is that 30-day readmission is often used in publications, which makes comparison more accurate; b) we wanted to avoid interference with new diseases that develop over time to really be able to focus on readmissions related to the initial diagnosis. We did not include this in the revised manuscript as we do not think that this is a crucial part for the understanding. However, if the editor feels that we should add a short paragraph to highlight these thoughts, we are certainly willing to do so.*

- The authors should stratify their findings concerning AMI as reason of readmission in the subtypes of AMI and by OPS codes for coronary interventions. These findings should also be incorporated in the rates of in-hospital mortality and might show differences over the time and by area (country).

*Unfortunately, this information is not available with sufficient accuracy. Due to countries having different coding versions, the coding is not sufficient across all countries and sites for this analysis. This is an important reason why we did not split diagnoses / readmissions in further details. Moreover, it would not change the frequency of readmissions due to causes other than the index event. Thus, the main message of the study is not influenced by this. Still, we agree with the reviewer that this would be interesting in a study that specifically addresses the issues of readmissions in AMI patients. However, this is beyond the scope of this study.*

- Table 4 shows the calculated odds-ratios of regression for probability of readmission with the same condition as initial hospitalisation. The authors should adjust the findings i.e. by area (country), length of stay and year of admission.

*We agree with the reviewer that this is interesting, which is why we did the multivariable regression analysis as depicted in table 4. Thus, the odds ratios provided are adjusted by all the variables included. Obviously, this was not clear which is why we added that the table contains the results of multivariable regression.*

Reviewer #2:

The issue of readmission rate in chronic disease is of outmost social and economic importance, but it has been often regarded from a disease-specific point of view. The Authors should be commended for their attempt to provide a picture of the possible paths patients with major chronic conditions often follow, using a huge amount of administrative data from all over the world.

*We would like to thank the reviewer for his/her positive comment on our study.*

While of interest, the value of the findings of the paper is limited from some major methodological issues, as reported below in details.

Major points

- The Authors state that they “included two acute conditions, i.e. AMI and bacterial pneumonia”. Indeed, stroke (acute?!) was also considered in the analysis. Although they were apparently included as they underly other chronic conditions, the Reviewer is rather concerned about this choice.

*We agree with the reviewer that stroke is also an acute event, based on an underlying chronic disease. We changed this accordingly in the revised manuscript.*

*Moreover, we understand the concern by the reviewer. In fact, we had some discussion about this point when designing the analysis. We decided to do so as we aimed to provide a broad picture of readmission of important different chronic diseases and that the underlying disease is most important for them. Also the other included conditions usually have acute events that lead to hospitalisation (e.g. acute decompensation of heart failure or CODP, occurrence of atrial fibrillation). In fact, the readmission rate of these three acute conditions were in line with the other conditions, supporting our choice. Therefore, we did not change the diseases included in the analysis. Nevertheless, we included the following paragraph to the limitation section:*

*“We included conditions that present with acute events (i.e. AMI, pneumonia, stroke) but are usually caused by a chronic underlying disease. Including these conditions might have influenced our results. However, readmission pattern of them were in line with the other more chronic conditions, supporting the assumption that the readmissions were mainly influenced by the underlying chronic condition rather than the acute event.”*

- Only readmissions at the same hospital could be recorded. Although the disease specific readmission rates are similar to those reported in the past (for example 7 vs 5% for heart failure, Fudim M et al. Aetiology, timing and clinical predictors of early vs. late readmission following index hospitalization for acute heart failure: insights from ASCEND-HF. Eur J Heart Fail. 2018;20:304-314), the whole number of readmission may have been significantly underestimated, especially as concerns those caused by different diseases (as patients may have been referred to different specialized hospitals).

*We fully agree with the reviewer which is why we included this discussion point in the original limitation section. In addition, we added the comparison with heart failure as an example as suggested by the reviewer.*

- Administrative and reimbursement issues may have significantly influenced the attribution of the readmission cause, thus possibly biasing the Authors’ findings.

*We fully agree with the reviewer. This is why we mention this point as our first limitation to our study. As also highlighted in one of the points raised by reviewer #1, there are pros and cons for the use of administrative data for this kind of analyses. For the purpose of our study, we are convinced that the inclusion of all cases of a specific hospital is an important pro, allowing the conclusions made.*

- How was time to first readmission related to the readmission cause?

*This is an interesting point. In fact, the time to readmission was exactly the same if comparing similar or different cause of readmission overall. There were small differences between the different original conditions. Due to the large number of patients, they obviously are all statistically significantly different, but of clinical relevance, only two are meaningful. We added this information to the manuscript in a separate paragraph of the Results section and added a figure (new figure 4).*

Minor points

- How do the Authors explain their observation that readmission rated have not changed significantly along the whole study period for most of the chronic conditions they have considered?

*We tend to disagree with the conclusion by the reviewer that there were no changes over time. In fact, there was a significant reduction in admission due to the same condition over time (1.3% per year) as indicated in table 4. As discussed, we hypothesise that this is related to the increase in age / complexity of chronic diseases over time.*

- Discussion should be shortened and focused on the main findings of the study.

*We agree in part with the reviewer and have shorten the discussion. We are also aware of the fact that our data are not sufficiently in depth to address all questions related to the topic. In order to stimulate the discussion and additional research in the field – both to better understand and to prevent readmissions – we deliberately discussed some issues where our results are more hypothesis generating than providing clear evidence.*

Reviewer #3:

Thank you for the opportunity to review this manuscript. It explored causes of admission and readmission in a unique population of multiple academic centers across the world. In a type of consortium 20+ centers have pooled administrative data for purposes of quality improvement research. IN this analysis the researchers found that in most cases the cause of readmission is different from the cause of admission the first time around. These findings have been previously described in a variable type of cohorts including administrative databases and clinical trial cohorts (see my last comment). Nevertheless this data remains very important and only underlines the need for a more “holistic” patient care (as the authors called it).

*We would like to thank the reviewer for the positive comment on our study.*

Comments:

- This study encompasses multiple countries. US policy on readmission reduction was cited but what about other countries? Do they have comparable initiatives?

*Although each healthcare system aims to reduce readmission rates, the US policy in this regard is rather unique. This is why we focused on discussing this in our manuscript. Given the fact that reduction in the length of the discussion is suggested by this and another reviewer, we have not extended our discussion in this point.*

- I am not sure I follow the difference between ICD and CCS. Aka Table 1 and 2.

*The ICD code defines diseases in more detail, where as the CCS summarises diseases in groups. It is referenced in the paper (see Methods section, ref 17)*

- It is important to understand where the ICD code for the primary diagnosis was derived from. Did the ICD code get obtained from the billing information, discharge notes etc? Only the code I the first position was used?

*Administrative dataset contain data that are used for national registers and are obtained from discharge notes. As mentioned in the manuscript, only the main diagnosis was used.*

- Found the amount of information provide in the figures to be somewhat overwhelming.

*We understand this notion by the reviewer. However, we are convinced that this is a strength of the manuscript as information is available if readers are interested in the details of our findings. This is why we did not reduce it.*

- Abbreviations in all tables and figures need to be presented in legends.

*We agree with the reviewer and changed this accordingly.*

- Information in the discussion section is repetitive. Reduce repetition and reduce in length.

*We agree in part with the reviewer and have shorten the discussion, particularly to avoid repetition unless required to understand the context of the according paragraph. We are also aware of the fact that our data are not sufficiently in depth to address all questions related to the topic. In order to stimulate the discussion and additional research in the field – both to better understand and to prevent readmissions – we deliberately discussed some issues where our results are more hypothesis generating than providing clear evidence.*

- Given that HF is a major contributor to the admission and readmission consider to discuss some of the already published literature on this topic. PMID: 27133201 and PMID: 29082629.

*We agree with the reviewer that HF is an important chronic condition. As also suggested by one of the other reviewers, we added one of the two suggested references to the manuscript, mentioning HF as example for comparison with our results.*
